# A Case Report on Ventriculoperitoneal Shunt Insertion Using a Novel Modification: The Receded Shunt Technique

**DOI:** 10.7759/cureus.86083

**Published:** 2025-06-15

**Authors:** Candice Marie Elizabeth D Tiongson, Joseph Erroll V Navarro, Oliver Ryan M Malilay

**Affiliations:** 1 Department of Surgery, Section of Neurosurgery, Jose R. Reyes Memorial Medical Center, Manila, PHL

**Keywords:** neuroplastics, neuro-reconstructive surgery, receded shunt, shunt erosion, shunting

## Abstract

A 36-year-old female with obstructive hydrocephalus secondary to adult-onset aqueductal stenosis and a right posterior parietal ventriculoperitoneal shunt presented with headaches and discomfort whenever the skin overlying her current valve was touched during hygiene and hair combing. She was diagnosed with a shunt malfunction and underwent removal of the previous shunt and insertion of a new shunt at the left frontal region using a novel method - the receded shunt technique. This involves creating a depression in the outer table where the shunt valve will be set to eliminate the apparent prominence of the valve. The advantages of this approach include better cosmesis, decreased skin tension, and increased patient comfort. Indeed, a simple modification such as this can provide a significant improvement to this age-old neurosurgical procedure.

## Introduction

Hydrocephalus is classified as either a communicating or non-communicating etiology. In adults, the most common cause of non-communicating hydrocephalus is aqueductal stenosis [1]. These patients present with headaches [2] or signs of increased intracranial pressure such as gait disturbances, visual impairments, or even a fatal decrease in sensorium [3].

Insertion of a ventriculoperitoneal shunt (VPS) can be a life-saving procedure, but it carries some widely reported risks: shunt malfunction, shunt infection, or, as highlighted in this study, shunt exposure. This may be seen anywhere from the cranium to the abdomen, mostly involving thin, tense skin [4].

A retrospective study in Egypt reports that among post-shunting complications, 23.3% had shunt exposure: 12.3% exposed valves and 10% exposed distal catheters [5].

Also, in another retrospective study in Singapore, in a total of 48 shunt failures, one patient had an exposed shunt valve, and another had an exposed catheter at the neck, both due to underlying infection [6].

Meanwhile, in a case series in Indonesia, a 2.3% incidence rate of shunt exposure, due to friable skin and malnutrition, was identified [7].

In the study of Bot et al. (2014), in the setting of malnourished patients with thin and friable skin, shunt exposure is commonly due to dehiscence. With this, they developed a subpericranial shunt valve placement to reinforce the skin closure tensile strength, which provided good wound closure to their patients.

Additionally, an obvious palpable prominent shunt valve leads to under-documented concerns in everyday living such as poor cosmesis, undue skin tension, and bothersome discomfort. One study at Johns Hopkins University developed the valve-agnostic cranial implant (VACI). This involves a customized artificial polyethylene implant with a receptacle receding the valve [8,9]. However, this involves additional costs and the use of resources.

This case report uses a similar concept with the receded shunt technique, a novel modification, countersinking the shunt valve below the outer table but without an artificial implant. Especially in the Philippines, where each hospitalization implies the consumption of already limited resources, this technique may reduce the chances of repeated reoperations and shunt revision due to shunt infection and exposure. It is important to note that this is a single-case study, and it is imperative to have more such studies in the future.

## Case presentation

A 36-year-old Filipino female consulted due to a one-month history of severe intermittent headaches, with no associated seizures, loss of consciousness, fever, or vomiting. She underwent a right posterior parietal VPS shunt insertion five years prior to consultation for adult-onset aqueductal stenosis, presenting with persistent headaches. On examination, she was awake, oriented, followed the conversation, and had isocoric brisk pupils, with no motor deficits; however, she had poor shunt rebound. Neurologic and ophthalmologic examinations were unremarkable. Additionally, she would occasionally complain of pain in the area of the shunt valve while combing her hair.

**Figure 1:**
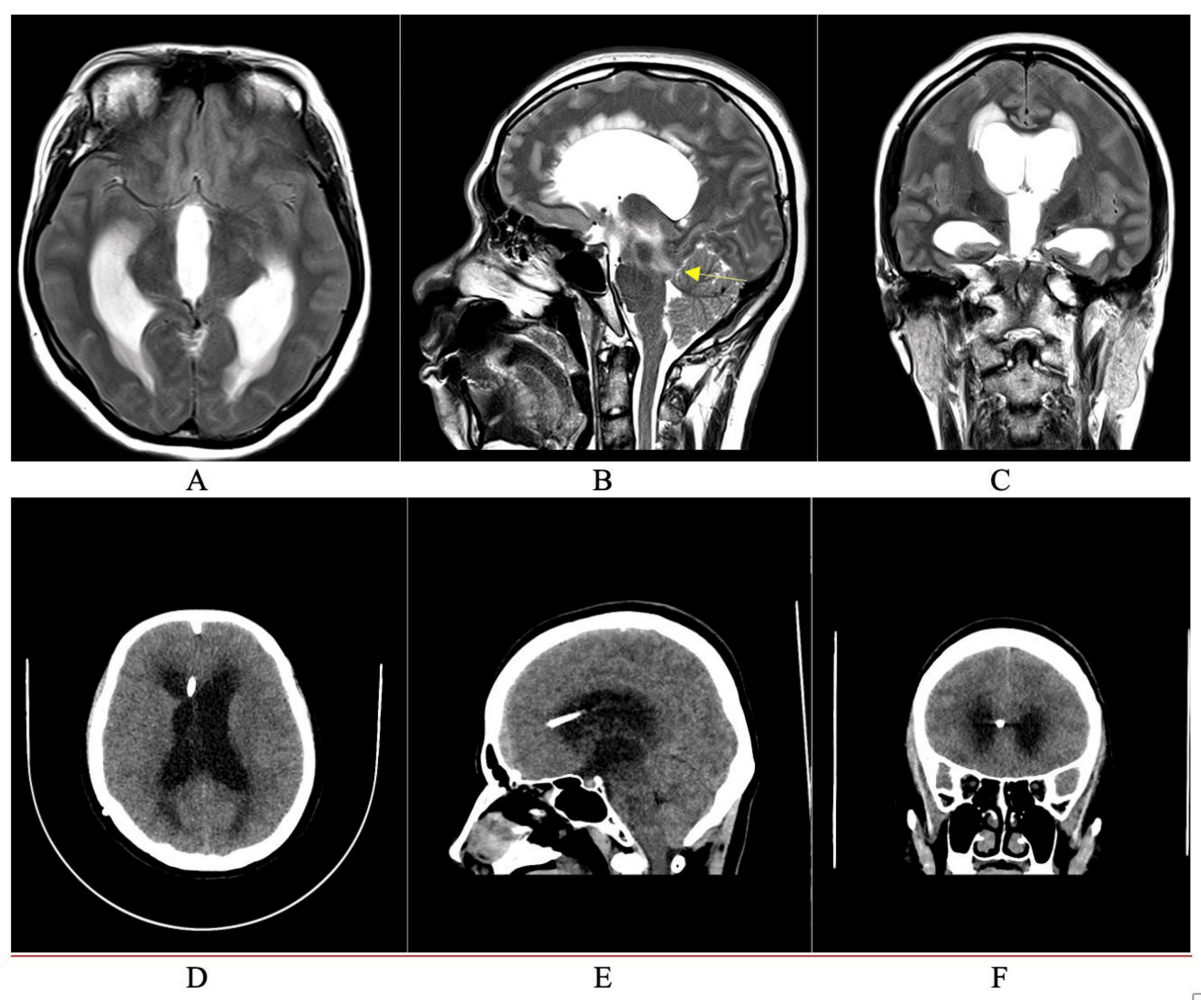
Cranial Imaging (A-C) Cranial MRI T2-weighted images showing obstructive hydrocephalus secondary to an adult-onset aqueductal stenosis (yellow arrow). A: Axial, B: Sagittal, C: Coronal, D-F: Axial, sagittal, and coronal cranial CT images, showing recurrence of the hydrocephalus, and the shunt tip abutting the right frontal lobe. D: Axial, B: Sagittal, C: Coronal.

On imaging (Figure 1), cranial magnetic resonance imaging (MRI) three years prior showed dilated lateral and third ventricles, with aqueductal stenosis and no apparent lesion. On cranial computed tomography (CT) done on admission, the ventricular tip abutted the right frontal parenchyma, with Evans’ Index of 0.29, with transependymal effusion. The previous shunt was removed, with the insertion of a new left frontal VPS using a programmable valve (Strata II ®™, Medtronic, Minneapolis, USA) [10]; this time countersinking the shunt valve below the outer table.

Surgical technique

A left frontal VPS insertion was done using a receded shunt technique in a tertiary government Brain and Spine institution in the Philippines.

Positioning

She was positioned supine, tilting the head to the right and elevating the neck and shoulder using a shoulder roll. The hair was clipped. The head, neck, and abdomen were prepared aseptically, followed by sterile draping.

Incision

**Figure 2:**
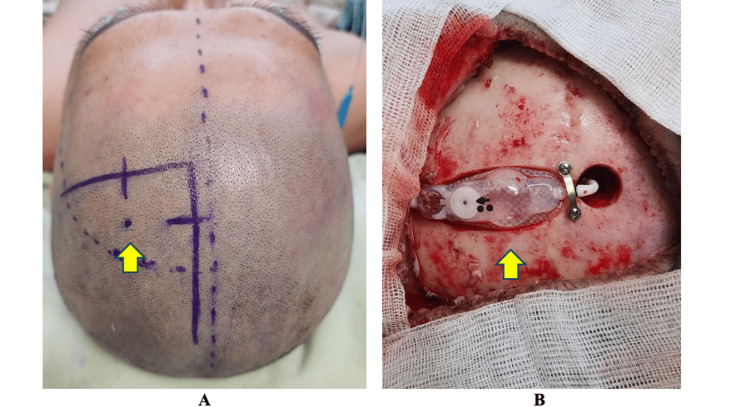
Incision and intraoperative images A: This image shows the outline of the triangular flap used in this procedure. A small dot denotes the left Kocher’s point. (yellow arrow) B: Final position of the valve after creating a calvarial pocket (yellow arrow) using a drill and securing the proximal and distal ends using titanium implants.

A triangular flap over Kocher’s point was planned for the ventricular end to facilitate space for the recession of the valve, each leg of the triangle measuring 10 cm. The base of the flap is positioned at the posterior parietal area (Figure 2A).

Shunt proper

The skin was incised, and a burrhole was placed 1 cm anterior to the coronal suture at the mid-pupillary line. The abdominal incision was placed 2 cm below the subcostal line and carried down into the peritoneal cavity. Next, the shape of the valve in its planned position, 2 cm posterior and lateral to the burrhole, was drawn on the skull using a sterile marker. Using a high-speed contouring drill, a calvarial pocket was formed where the valve was placed, deep enough to recess the valve at least 75% below the outer table. To prevent kinking due to an abrupt step-off, a tapered tunnel was drilled for the junction of the valve and distal catheter. The tunneling of the peritoneal catheter and insertion of the ventricular catheter proceeded in a typical manner. The valve was then attached to the proximal and distal end and secured using screws and curved titanium plates on its proximal and distal ends. The shunt patency was tested, and the abdominal end was inserted into the intraperitoneal cavity and subsequently secured. The final configuration of the valve is shown in Figure 2B.

The Medtronic Strata II ®™ valve is programmable, with performance levels adjusted using a magnetic tool. Prior to closure, the performance level is set to the highest (2.5) to avoid overdrainage. After the insertion of the new shunt, the previous shunt was removed. No complications ensued in the operation.

**Figure 3:**
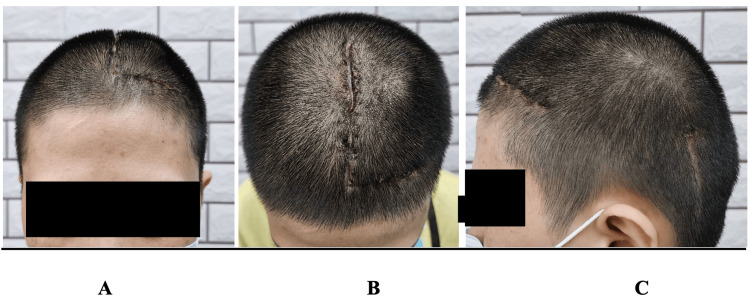
Postoperative site Anterior (A), superior (B), and lateral (C) views of the postoperative site two weeks after the procedure. The incision was done to allow the surgeon to have space to recede the valve. The shunt valve shows little visible impression on the scalp.

She had an unremarkable postoperative course and was discharged after a few days. On follow-up, the skin over the valve was less obvious than usual shunts (Figure 3). She then expressed satisfaction with the appearance and comfort despite having a shunt as compared to earlier, which was maintained even during follow-up after six months and one year.

## Discussion

The receded shunt technique offers many advantages over the conventional shunt technique. Similar to the VACI, it offered improved cosmesis, decreased tension, and greater comfort [8,9].

For cosmesis, this technique eliminated the apparent bump that a shunt valve leaves on the scalp. More than the physical appearance, it addressed the psychosocial effects of having an obvious shunt valve. Though the procedure calls for the concealment of the device, its functionality is preserved. Just as with the VACI, an allowance of 1 mm height or 25% is provided to allow palpability, should a shunt tap be needed [8].

It also decreases the skin tension over the valve during skin closure, for better wound healing and decreased chances of infection. Prolonged pressure on the overlying skin leads to ischemia, necrosis, and ultimately skin breakage. The most common area of shunt exposure is over the valve - the point of highest tension [11].

Lastly, it eliminated the discomfort, tenderness, and irritation over the valve on combing one’s hair, bathing, and other activities of daily living. There is still a paucity of literature that documents patients’ comfort and satisfaction after a shunting procedure.

In contrast to the VACI, this procedure eliminated the additional implant, decreasing the chances for additional adverse reactions to porous polyethylene, although risks are very low [12], decreasing the financial burden.

However, an additional step in the procedure increases the OR time, and if not planned efficiently, might add to the risk of infection. Second, a larger incision to recede the flat bottom valve may not be aesthetically pleasing for the patient. Given that, this procedure is not limited to a flat-bottom-type valve; it can be tailored to any type. If a burrhole valve were to be used, a smaller incision may be used since the area of recession makes use of the burrhole itself, and without the need for additional calvarial surface area.

Furthermore, more trials and literature searches can be conducted to evaluate this technique.

## Conclusions

Indeed, the receded shunt technique offers better cosmesis, decreased skin tension, and increased comfort while preserving the functionality of the device. For this patient, not only did it alleviate signs of increased intracranial pressure and shunt malfunction, but it also addressed concerns that are not usually emphasized: comfort and cosmesis. With this, this study can then potentially open opportunities to decrease the risks of shunt exposure, decreasing the need for repeated shunt revisions.
